# More or less of me and you: self-relevance augments the effects of item probability on stimulus prioritization

**DOI:** 10.1007/s00426-021-01562-x

**Published:** 2021-07-29

**Authors:** Saga L. Svensson, Marius Golubickis, Hollie Maclean, Johanna K. Falbén, Linn M. Persson, Dimitra Tsamadi, Siobhan Caughey, Arash Sahraie, C. Neil Macrae

**Affiliations:** 1 School of Psychology, King’s College, University of Aberdeen, Aberdeen, AB24 3FX Scotland, UK; 2 School of Psychology, University of Plymouth, Plymouth, England UK

## Abstract

Self-relevance exerts a powerful influence on information processing. Compared to material associated with other people, personally meaningful stimuli are prioritized during decision-making. Further exploring the character of this effect, here we considered the extent to which stimulus enhancement is impacted by the frequency of self-relevant versus friend-relevant material. In a matching task, participants reported whether shape-label stimulus pairs corresponded to previously learned associations (e.g., triangle = self, square = friend). Crucially however, before the task commenced, stimulus-based expectancies were provided indicating the probability with which both self- and friend-related shapes would be encountered. The results revealed that task performance was impacted by the frequency of stimulus presentation in combination with the personal relevance of the items. When self- and friend-related shapes appeared with equal frequencies, a self-prioritization effect emerged (Expt. 1). Additionally, in both confirmatory (Expt. 2) and dis-confirmatory (Expt. 3) task contexts, stimuli that were encountered frequently (vs. infrequently) were prioritized, an effect that was most pronounced for self-relevant (vs. friend-relevant) items. Further computational analyses indicated that, in each of the reported experiments, differences in performance were underpinned by variation in the rate of information uptake, with evidence extracted more rapidly from self-relevant compared to friend-relevant stimuli. These findings advance our understanding of the emergence and origin of stimulus-prioritization effects during decisional processing.

**Supplementary Information:**

The online version contains supplementary material available at 10.1007/s00426-021-01562-x.

## Introduction

Reflecting the fundamental role that self-relevance exerts during information processing, recent years have witnessed a burgeoning interest in the degree to which the personal significance of otherwise arbitrary material, most notably geometric shapes, influences decision-making (Humphreys & Sui, 2016; Sui & Humphreys, 2015, 2017). Extending earlier research highlighting the memorial advantages of stimulus relevance (Symons & Johnson, 1997), self-prioritization—whereby decision-making is facilitated for self-relevant (vs. other-relevant) information—has been documented across a wide range of task contexts (e.g., Frings & Wentura, 2014; Macrae et al., 2018; Mattan et al., 2015; Moradi et al., 2015; Payne et al., 2017; Schäfer et al., 2015, 2016; Sui et al., 2012; Woźniak & Knoblich, 2019). Driving these effects, it has been claimed, is a mind that is exquisitely receptive to self-relevant inputs, such that they are enhanced during decisional processing (Humphreys & Sui, 2016; Sui & Humphreys, 2015, 2017).^1^ Extending work on this core social-cognitive topic, here we considered the extent to which the frequency of stimulus presentation influences the processing of material pertaining to the self and others (Falbén et al., 2020; Sui et al., 2014).

## The self and stimulus prioritization

In a complex social world, it makes functional sense for minds to be responsive to material paired with targets other than the self. Although self-relevant stimuli unquestionably loom large in many settings (Constable et al., 2011, 2014), there are also countless occasions in which it would be foolhardy to prioritize these items (e.g., focusing on one’s already topped up wine glass when the boss requests a beverage). Put simply, were currently goal-irrelevant stimuli prioritized by dint of their personal significance, information processing (and response selection) would rapidly grind to a halt. To service a flexible behavioral repertoire, stimulus prioritization must be sensitive to a range of influences, of which self-relevance is but one (Reuther & Chakravarthi, 2017; Schäfer et al., 2017; Wade & Vickery, 2017). Thus, in principle, there should be a range of factors that trigger stimulus prioritization during decisional processing.

Developing this line of reasoning, Falbén et al., (2020) proposed that stimulus prioritization is likely influenced by information that has been overlooked in typical investigations of self-bias (Golubickis et al., 2018; Sui et al., 2012), but is routinely present and utilized in life outside the laboratory; prior knowledge regarding the probability of encountering person-related stimuli in various settings. Before reconnoitring a partner’s walk-in closet, for example, one would anticipate encountering a multitude of their garments and perhaps only a few items of one’s own (and vice versa for one’s own closet). When available and applicable, expectations wield a powerful influence on perception and judgment (Bar, 2007; Barbey & Sloman, 2007; Pennycook & Thompson, 2012). Specifically, processing is facilitated for expected compared to unexpected material, with prior expectations about the prevalence of stimuli impacting the operations that support decision-making (De Loof et al., 2016; Domenech & Dreher, 2010; Dunovan et al., 2014).

To explore the effects of prior beliefs on self-prioritization, Falbén and colleagues adopted an object-ownership paradigm (Golubickis et al., 2018). As psychological extensions of the self, one’s possessions are advantaged during stimulus appraisal (Morewedge & Giblin, 2015; Pierce et al., 2003). Compared to other people’s belongings, personal possession confers a processing benefit during object detection, classification, and recollection—the so-called self-ownership effect (Constable et al.Cunningham et al., 2008; Golubickis et al., 2018, 2019). Exploiting this robust phenomenon, Falbén et al. (2020) demonstrated that stimulus prioritization was moderated by the frequency with which self- and friend-related items were encountered during an object-ownership task. When no information about the prevalence of stimulus presentation was available, replicating previous research, self-owned objects were classified more rapidly than items owned by a friend (Golubickis et al., 2018), a self-prioritization effect that was abolished when participants were informed that self- and friend-owned objects were equally likely to be encountered during the task. Furthermore, in both confirmatory and dis-confirmatory task contexts—regardless of ownership—frequently (vs. infrequently) presented items elicited stimulus prioritization. Adopting a drift diffusion model (DDM) analysis to elucidate the origin of this effect (Ratcliff et al., 2016; Voss et al., 2013), stimulus prioritization was traced to the operation of a response bias (Falbén et al., 2020). Specifically, less evidence was required when judging frequently compared to infrequently presented stimuli (De Loof et al., 2016; Dunovan et al., 2014).

## Stimulus frequency and decision-making

Falbén et al.’s (2020) findings were noteworthy for a number of reasons. At least in the context of an object-ownership task, they demonstrated the flexibility of stimulus prioritization and traced decisional bias to differences in the evidential requirements of response generation (Golubickis et al., 2018, 2020; Reuther & Chakravarthi, 2017; Schäfer et al., 2017; Wade & Vickery, 2017). Notwithstanding these observations however, critical issues remain. Three in particular merit consideration. First, although ownership has proved a productive arena for exploring the effects of self-relevance on decision-making (Constable et al., Falbén et al., 2019; Golubickis et al., 2018, 2019; Truong et al., 2017), stimulus prioritization has been most strongly linked with matching tasks in which participants judge the accuracy of previously formed shape-label pairings (Sui et al., 2012, 2014). Thus, it is unclear if the effects observed when participants respond to expected or unexpected objects associated (i.e., owned by) with the self and a friend also extend to tasks in which geometric shapes serve as proxies for these targets (Humphreys & Sui, 2016; Sui & Humphreys, 2015).

Second, Falbén et al.’s (2020) demonstration of the elimination of self-bias is surprising given the reported ubiquity of this effect (Reuther & Chakravarthi, 2017; Schäfer et al., 2017; Sui & Humphreys, 2017; Sui et al., 2014; Wade & Vickery, 2017). For example, prior to the performance of a matching task, Reuther and Chakravarthi (2017) introduced a training phase in which error-free learning was equated for all the shape-label stimulus pairs. Despite this extensive pre-task preparation, self-prioritization persisted. Given life-long experience dealing with personally relevant material, this bias probably derives from the enhanced accessibility of self-shape (vs. friend-shape) relations in working memory against which stimuli must be compared to perform the matching task (Caughey et al., 2021; Constable et al., 2019a, 2019b; Reuther & Chakravarthi, 2017; Wade & Vickery, 2017). In addition, a central premise of Sui and Humphrey’s Self-Attention Network (SAN) model is that self-relevance facilitates the processing of personally meaningful inputs through the interplay of top–down (i.e., self-activation) and bottom–up (i.e., allocation of attention) processes in an obligatory manner (Humphreys & Sui, 2016; Sui & Humphreys, 2015, 2017; Sui et al., 2013). Collectively, these observations suggest that the abolishment of self-bias would not be anticipated.

Third, it is possible that Falbén et al.’s (2020) behavioral and modeling findings derived from specific characteristics of the ownership task that was adopted. Based on previous research (De Loof et al., 2016; Dunovan et al., 2014), a key element of Falbén et al.’s paradigm was that self-owned and friend-owned items were presented with different frequencies (e.g., self-frequent vs. friend-frequent). What of course this means in the context of an object-classification task is that one of the response keys (i.e., self-owned or friend-owned) was used more often than the other. Accordingly, it is possible that potent response-related expectancies masked the contribution that stimulus-based processes (i.e., rate of evidence gathering) play during decision-making (Golubickis et al., 2017, 2020; Humphreys & Sui, 2016; Sui & Humphreys, 2015, 2017; White & Poldrack, 2014).

Contrasting Falbén et al.’s (2020) methodology, a useful feature of matching tasks is that participants respond to shape-label pairings that correspond to previously learned associations (e.g., triangle = you, square = friend) on half the experimental trials, but mismatch these associations (e.g., square = you, triangle = friend) on the remaining trials (Sui et al., 2012). As the response options (i.e., matching vs. nonmatching) are used equally often during the task and do not explicitly reference self or other, this therefore raises the possibility of manipulating stimulus frequencies without creating a bias for either self- or friend-related responses. In this way, shape-label matching tasks afford the ability to explore stimulus prioritization in a task context absent response-related expectancies, thus are better able to probe the role that stimulus-based processes may play during decision-making (Sui et al., 2012).

To date, only a single investigation by Sui et al. (2014) has considered how the frequency of presentation of items pertaining to the self and others (i.e., mother & stranger) influences self-prioritization in a shape-label matching task. Motivating this research was the observation that processing is routinely impacted by the probability with which different classes of stimuli appear, such that performance is enhanced for frequently (vs. infrequently) presented items (e.g., Logan et al., 1984; Milligan & Lupiáñez, 2005; Schmidt, 2013; Schmidt & Besner, 2008). Interestingly, however, across a series of studies, Sui et al. (2014) demonstrated that self-prioritization was indifferent to the frequency of stimulus presentation, with self-bias emerging even when self-relevant (vs. other-relevant) items were presented on only a minority of trials (i.e., self-prioritization superseded the effects of stimulus frequency). In addition, although stimuli relating to mother yielded a prioritization effect when these items predominated during the task, this processing benefit only emerged when stranger was the target of comparison (i.e., mother < stranger). When contrasted with responses to self-relevant items, performance was equivalent (i.e., mother = self). Albeit in a different task context, these findings conflict with Falbén et al.’s (2020) demonstration that, regardless of the target of association, frequently (vs. infrequently) encountered stimuli were prioritized during decision-making.

### The current research

Noting these inconsistencies in the extant literature (Falbén et al., 2020; Sui et al., 2014), here we considered the extent to which the likelihood of encountering self- and friend-related items (i.e., geometric shapes) during a shape-label matching task influences stimulus prioritization. Following Sui et al. (2012), participants initially formed shape-label associations, after which they judged whether stimulus pairs matched or mismatched the previously forged relations. Crucially, however, before the matching task began, expectancies about the prevalence with which self- and friend-related shapes would be encountered were provided (Falbén et al., 2020), beliefs that ultimately were confirmed (i.e., Expt. 1 & 2) or disconfirmed (i.e., Expt. 3) by the actual frequency of stimulus presentation.

The goal of our first experiment was to establish the influence that self-relevance per se exerts during shape-label matching. As such, participants were told to expect an equivalent number of self- and friend-related items to be presented. Unlike Falbén et al., (2020), absent the operation of self-other response-related expectancies—and reflecting the strength of self-shape associations in working memory—a self-prioritization effect was expected to emerge on matching trials under these conditions (Reuther & Chakravarthi, 2017; Sui et al., 2012; Wade & Vickery, 2017).^2^ To identify the processes underlying task performance (i.e., stimulus and/or response biases), a drift diffusion model (DDM) analysis was conducted on the data as this approach has been applied successfully in previous work exploring the origins of stimulus prioritization (Falbén et al., 2020; Golubickis et al., 2017, 2018, 2020). Replicating previous research that has used shape-label matching tasks to probe self-bias (Golubickis et al., 2017, 2020), we expected self-prioritization to be underpinned by differences in the rate of information uptake during decisional processing. Specifically, information would be extracted more rapidly from self-related compared to friend-related items.

## Experiment 1: equivalent context

### Method

#### Participants and design

Twenty undergraduates (14 female, *M*_age_ = 19.32, *SD* = 5.08) took part in the experiment. All participants had normal or corrected-to-normal visual acuity. Informed consent was obtained from participants prior to the commencement of the experiment and the protocol was reviewed and approved by the Ethics Committee at the School of Psychology, University of Aberdeen. The experiment had a 2 (Shape Association: self or friend) X 2 (Matching Condition: matching or nonmatching) repeated-measures design. The smallest effect size that could be detected for a within-participants two-way interaction with a sample of 20 participants, 50 observations per experimental condition, and 80% power, was estimated to be *d* = 0.38 (PANGEA v0.2).

#### Stimulus materials and procedure

Participants arrived at the laboratory individually, were greeted by an experimenter, seated at a desktop computer, and informed that the study comprised a matching task featuring geometric shapes that represented them (i.e., self) and their best friend (Sui et al., 2012). The experimenter then explained that participants would be presented with a shape and a label on the computer screen and their task was to indicate, via a button press as quickly and accurately as possible, whether the shape-label pair matched or non-matched the previously formed associations. Responses were given using two keys on the keyboard (i.e., N and M). Key-response mappings were counterbalanced across participants and the labels ‘match’ and ‘non-match’ located above the relevant response key. Participants were informed that during the task, an equal number of self-related and friend-related shapes (i.e., 50% self & 50% friend) would be presented. Next, participants were told that the computer would randomly assign one geometric shape to denote them and another shape to denote their friend. They then pressed spacebar on the keyboard and were shown a screen indicating which geometric shapes designated self and friend, respectively (e.g., you = square, friend = triangle). The assignment of shapes to self and friend was counterbalanced across the sample and the shapes were not presented during this phase of the task (Sui et al., 2012).

Each trial began with the presentation of a central fixation cross for 1000 ms, followed by the pairing of a shape above, and a label below, the fixation cross, respectively, for 100 ms. After each shape-label pairing was presented, the screen turned blank until participants reported the accuracy of the association. The stimuli comprised two white images of geometric shapes (i.e., triangle & square) on a grey background. Images were 138 × 138 pixels in size. The labels designating the participant and their friend were ‘You’ or ‘Friend’ and appeared below the shapes.

Participants initially performed ten practice trials, followed by a block of 200 experimental trials. Half of the trials displayed a matching association and half a nonmatching shape-label pairing (i.e., the shape did not correspond with the label; see Sui, et al., 2012, 2013, 2014). Thus, there were 50 trials in each condition (i.e., self-matching, self-nonmatching, friend-matching, and friend-nonmatching). A self-matching trial displayed an image of a square (or triangle, depending on the counterbalancing) above the fixation cross, together with the label ‘You’ underneath the fixation cross. The order in which the trials were presented was randomized. On completion of the task, participants were debriefed, thanked, and dismissed.

## Results

### Response time

**Fig. 1:**
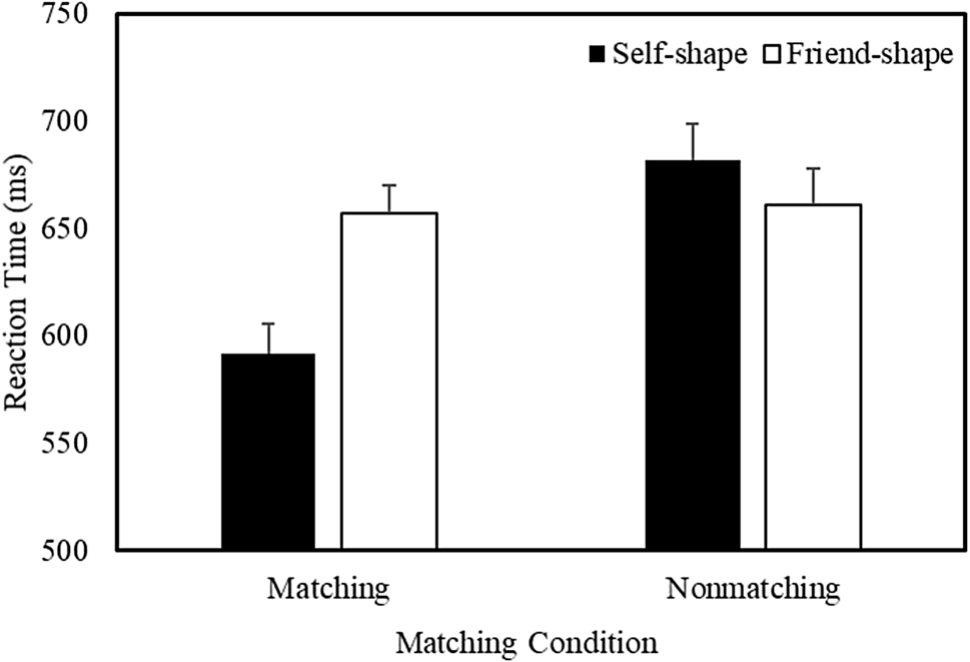
Mean response time as a function of Shape Association and Matching Condition (Expt. 1—Equivalent Context). Error bars represent + 1 SEM

Responses faster than 200 ms were excluded from the analysis (Sui et al., 2012), eliminating less than 1% of the overall trials. A multilevel model analysis was used to examine the correct response time (RT) and accuracy data (see Table S1 in the Supplementary Material for a listing of all the treatment means). Analyses were conducted using the R package ‘lmer4’ (Pinheiro et al., 2015). Shape Association and Matching Condition were treated as categorical fixed effects, and participants as a crossed random effect (Judd et al., 2012). The analysis of the RTs yielded main effects of Shape Association (*b* =  − 10.88, *SE* = 3.43, *t* =  − 3.19, *p* = 0.001, *R*^2^ = 0.10), Matching Condition (*b* =  − 24.02, *SE* = 3.41, *t* =  − 10.37, *p* < 0.001, *R*^2^ = 0.12), and a significant Shape Association X Matching Condition (*b* =  − 22.45, *SE* = 3.41, *t* =  − 6.58, *p* < 0.001, *R*^2^ = 0.13) interaction. Further analysis of the interaction (Fig. 1) revealed that, during matching trials, responses were faster to self-related compared to friend-related stimuli (*b* =  − 33.51, *SE* = 4.75, *t* =  − 7.06, *p* < 0.001, *R*^2^ = 0.13). In contrast, during nonmatching trials, responses were faster to friend-related than to self-related items (*b* = 11.22, *SE* = 4.91, *t* = 2.29, *p* = 0.02, *R*^2^ = 0.11).

### Accuracy

**Fig. 2:**
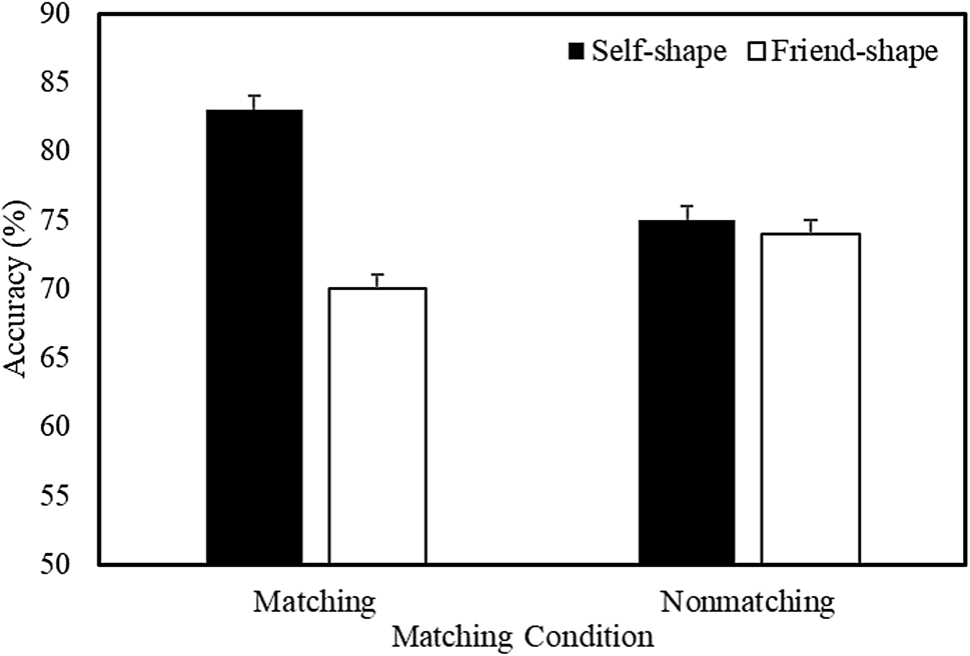
Mean accuracy as a function of Shape Association and Matching Condition (Expt. 1—Equivalent Context). Error bars represent + 1 SEM

A multilevel logistic regression analysis on the accuracy of responses revealed a main effect of Shape Association (*b* = 0.21, *SE* = 0.04, *z* = 5.04, *p* < 0.001, *R*^2^ = 0.08) and a significant Shape Association X Matching Condition (*b* = 0.17, *SE* = 0.041, *z* = 4.16, *p* < 0.001, *R*^2^ = 0.09) interaction. Further analysis of the interaction (Fig. 2) revealed that, during matching trials, accuracy was higher for self-related compared to friend-related stimuli (*b* = 0.38, *SE* = 0.06, *z* = 6.31, *p* < 0.001, *R*^2^ = 0.09). During nonmatching trials, no significant difference in accuracy was observed.

### Drift diffusion modeling

A DDM analysis was used to explore the processes underpinning task performance. Previous computational investigations of self-prioritization have shown that, during a shape-label matching task, there are several decisional processes that drive self-bias. Specifically, self-relevance modulates the rate of information uptake and elicits a response bias toward matching (vs. nonmatching) judgments (Golubickis et al., 2017, 2020). Given these findings, the current modeling analysis examined the extent to which stimulus- and response-related processes underpin shape-label matching (White & Poldrack, 2014).

The DDM uses both response latency and accuracy to estimate the latent cognitive processes associated with task performance and how they unfold over time (Ratcliff et al., 2016). During binary decision-making (e.g., is a shape-label pairing matching or nonmatching), evidence is continuously gathered from a stimulus until one or other response threshold has been reached. The benefit of sequential sampling models, such as the DDM, lies in the ability to identify the stimulus and/or response biases that underpin task performance (Ratcliff et al., 2016; Voss et al., 2013; White & Poldrack, 2014). In this respect, the DDM comprises four parameters that describe decisional processing. First, drift rate (*v*) estimates the quality and speed of evidence sampling (i.e., larger *v* = faster information uptake). This component represents noisy information accumulation during decision-making, and thus is treated as a measure of perceptual efficiency. The second parameter is boundary separation (*a*). It quantifies the space (i.e., distance) between the two response thresholds, and thus refers to the amount of information required before a judgment is made. Large values of *a* represent a conservative and cautious decision-making style, whereas small values signal a more liberal, less careful approach. Next, between the two response boundaries, the starting point (*z*) specifies the position at which the noisy information sampling process begins. In situations in which *z* is not centred between the boundaries, the starting point represents a response bias in favor of the nearer boundary (i.e., variation in the evidential requirements of response selection). Finally, all processes that do not contribute to decision-making (e.g., stimulus encoding, response execution) are described by the non-decision time parameter (*t*_*0*_).

To elucidate the origins of self-prioritization, a hierarchical drift diffusion model (HDDM) analysis was conducted on the data (Vandekerckhove et al., 2011). HDDM is an open-source Python toolbox for the hierarchical Bayesian computation of DDM decisional components (Wiecki et al., 2013). The HDDM treats model parameters for individual participants as random samples constrained by group-level distributions (Vandekerckhove et al., 2011). Following previous research (Golubickis et al., 2017, 2020), the models were response coded (i.e., upper threshold = matching response, lower threshold = nonmatching response). Four models were estimated for comparison (see Table 1). The first two models allowed the drift rate (*v*) and starting point (*z*) to vary as a function of Matching Condition (i.e., matching vs. nonmatching). These comprise the two simplest models available in a shape-label matching task and consider whether performance is underpinned by differences in the rate of information uptake between matching and nonmatching trials and/or response-related processes indicative of a confirmatory matching-bias (Golubickis et al., 2017, 2020). Critically, models 1 and 2 assumed no difference in the rate of information uptake as a function of Shape Association. The third model allowed the drift rate (*v*) to vary as a function of Shape Association (i.e., self vs. friend) and Matching Condition, while the starting point (*z*) was not estimated (*z* = 0.50, no bias). This parameterization examined whether decision-making was underpinned solely by differences in the efficiency of stimulus processing. Finally, model 4 allowed the drift rate (*v*) to vary as a function of Shape Association and Matching Condition and the starting point (*z*) to vary between the response thresholds. This parameterization considered whether task performance was facilitated by increased information uptake, with response-related processes contributing toward a matching-bias. Previously, this parameter setup has been found to be best fitting in shape-label matching tasks (i.e., default model; Golubickis et al., 2017, 2020). All four models estimated the inter-trial variability for drift rate (*sv*), non-decision time (*st*), and starting point (*sz*).

**Table 1:** Deviance information criterion (DIC) for each model (Expt. 1)

Model	Shape association	Matching condition	DIC
1	–	*v*	3037
2	–	*z, v*	3010
3	*v*	*v*	2985
4	*v*	*z, v*	2953

*v* = drift rate, *z* = starting point

For each model, 10,000 Markov Chain Monte Carlo (MCMC) samples (1,000 burn-in) were simulated. Due to its computational efficiency and widespread use when using hierarchical models, the Deviance Information Criterion (DIC) was adopted as a measure of fit for the model comparisons (Spiegelhalter et al., 1998). Lower DIC values indicate greater fit as they favor models with the least number of parameters and highest likelihood. As can be seen in Table 1, in line with previous research, model 4 was the best fit (Golubickis et al., 2017, 2020). Analysis of the posterior distributions revealed that task performance was underpinned by differences in information uptake (drift rate, *v*) and the evidential requirements of response generation (starting point, *z*, Table 2). During matching trials, there was extremely strong evidence that information uptake (i.e., drift rate) was faster for self-relevant compared to friend-relevant stimuli (negative drift rates were first multiplied by − 1) (*p*_Bayes_[self > friend] < 0.001).^3^ No evidence for a difference in drift rates was observed during nonmatching trials (*p*_Bayes_[self > friend] = 0.409). In addition, comparing the starting point value (*z*) with no bias (*z* = 0.50) yielded extremely strong evidence of a response bias in favor of matching (vs. nonmatching) judgments (*p*_Bayes_[bias > 0.50] < 0.001).

**Table 2:** Parameter means and the upper (97.5q) and lower (2.5q) quantiles of the best fitting model (Expt. 1)

Diffusion model parameter	Mean	Quantile	
		2.5q	97.5q
a	0.957	0.88	1.044
v_matching-self_	1.744	1.294	2.222
v_matching-friend_	0.79	0.572	1.037
v_nonmatching-self_	− 1.372	− 1.897	− 0.854
v_nonmatching-friend_	− 1.399	− 1.915	− 0.846
z	0.544	0.524	0.567
t_0_	0.437	0.41	0.465
st	0.446	0.418	0.475
sv	0.151	0.005	0.413
sz	0.304	0.085	0.450

*a* threshold separation, *v* drift rate, *z* starting point, *t*_*0*_ non-decision time, *sv* inter-trial variability in drift rate, *st* inter-trial variability in non-decision time, *sz* inter-trial variability in starting point

## Discussion

Supporting our hypothesis, under conditions in which equivalent frequencies of self- and friend-related shapes were presented, a self-prioritization effect emerged. Specifically, responses on shape-label matching trials were faster and more accurate to self-related compared to friend-related items (cf. Falbén et al., 2020). In addition, an HDDM analysis revealed that self-prioritization was underpinned by a stimulus bias. Notably, information uptake (i.e., drift rate) was faster for self-relevant than friend-relevant stimuli (Golubickis et al., 2017, 2020). Although failing to replicate Falbén et al.’s (2020) elimination of self-prioritization when self- and friend-related items were expected to appear equally often in an object-ownership task, the current results resonate with prior work that has explored self-bias using shape-label matching tasks (Sui et al., 2012, 2014). As attention must explicitly be directed to the self-relevance (or otherwise) of shape-label relations to perform the matching task successfully (Caughey et al., 2021), the strongest associative linkages in working memory exert the greatest influence on task performance (Reuther & Chakravarthi, 2017). Accordingly, when stimulus frequencies (i.e., self & other) are equivalent—and potent response-related expectancies are absent (cf. Falbén et al., 2020)—a self-prioritization effect emerges.

Extending the scope of the current investigation, in our next experiment, we varied the frequency with which self- and friend-related shapes were encountered during the shape-label matching task (Falbén et al., 2020; Sui et al., 2014). Specifically, prior to the commencement of the task, participants were told to expect a higher frequency of either self-shapes or friend-shapes to be presented (i.e., 75% vs. 25%), expectancies that were confirmed by the actual prevalence of stimulus presentation. Based on the results of Experiment 1 (i.e., default bias toward self-relevant stimuli), we expected that frequently (vs. infrequently) encountered stimuli would be prioritized during decisional processing (cf. Sui et al., 2014), but this effect would be larger for self-relevant compared to friend-relevant items (cf. Falbén et al., 2020). As previously, an HDDM analysis was conducted to identify the processes driving task performance, with a stimulus bias hypothesized to underpin item prioritization.

## Experiment 2: confirmatory context

### Method

#### Participants and design

Twenty-four undergraduates (16 female, *M*_age_ = 19.72, *SD* = 2.33) took part in the experiment. All participants had normal or corrected-to-normal visual acuity. Informed consent was obtained from participants prior to the commencement of the experiment and the protocol was reviewed and approved by the Ethics Committee at the School of Psychology, University of Aberdeen. The experiment had a 2 (Stimulus Frequency: self-frequent or friend-frequent) X 2 (Shape Association: self or friend) X 2 (Matching Condition: matching or nonmatching) repeated-measures design. The smallest effect size that could be detected for a within-participants three-way interaction (for Experiments 2 & 3) with a sample of 24 participants, 50 observations per experimental condition, and 80% power, was estimated to be *d* = 0.24 (PANGEA v0.2).

#### Stimulus materials and procedure

Participants arrived at the laboratory individually, were greeted by an experimenter, seated at a desktop computer, and informed that the study comprised a matching task featuring geometric shapes that represented them (i.e., self) and their best friend (Sui et al., 2012). The task closely followed Experiment 1, but with an important modification. Participants were told that they would complete two blocks of trials: one with a higher frequency of self-related shapes (i.e., 75% self & 25% friend), and one with a higher frequency of friend-related shapes (i.e., 75% friend & 25% self). Block order was counterbalanced across the sample and participants were reminded whether the self-shape or friend-shape would predominate prior to the respective block. Participants initially performed ten practice trials, after which the main task commenced. Each block comprised 200 trials. The self-frequent block consisted of 100 matching and 100 nonmatching trials. Of these, 150 comprised self-related shape-label pairings, with 75 matching and 75 nonmatching trials. The remaining 50 trials comprised friend-related shape-label pairings, with 25 matching and 25 nonmatching trials. The friend-frequent block had an identical trial structure, but with friend-related shape-label pairings presented most frequently. The order of the trials was randomized in each block. On completion of the task, participants were debriefed, thanked, and dismissed.

## Results

### Response time

**Fig. 3:**
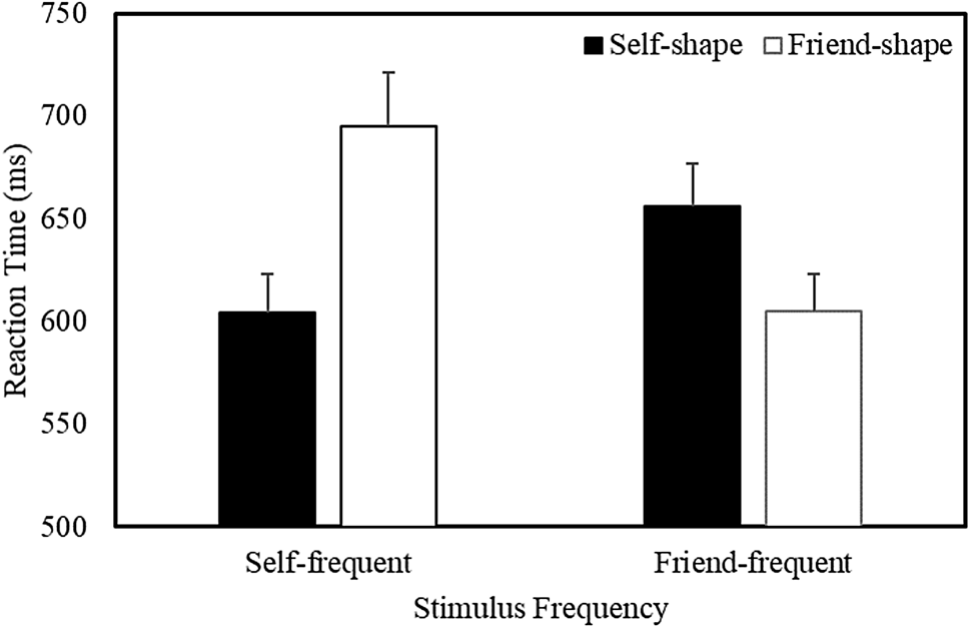
Mean response time as a function of Stimulus Frequency and Shape Association (Expt. 2—Confirmatory Context). Error bars represent + 1 SEM

Responses faster than 200 ms were excluded from the analysis (Sui et al., 2012), eliminating less than 2% of the overall trials. A multilevel model analysis was used to examine the correct RT and accuracy data (see Table S2 in the Supplementary Material for a listing of all the treatment means). Stimulus Frequency, Shape Association, and Matching Condition were treated as categorical fixed effects, and participants as a crossed random effect. The analysis of RTs yielded main effects of Stimulus Frequency (*b* = 9.13, *SE* = 2.48, *t* = 3.67, *p* < 0.001, *R*^2^ = 0.19), Shape Association (*b* =  − 9.64, *SE* = 2.48, *t* =  − 3.88, *p* < 0.001, *R*^2^ = 0.19), and Matching Condition (*b* =  − 25.76, *SE* = 2.49, *t* =  − 10.37, *p* < 0.001, *R*^2^ = 0. 21), and significant Stimulus Frequency X Shape Association (*b* =  − 35.12, *SE* = 2.49, *t* =  − 14.13, *p* < 0.001, *R*^2^ = 0.22) and Shape Association X Matching Condition (*b* =  − 5.71, *SE* = 2.49, *t* =  − 2.30, *p* = 0.02, *R*^2^ = 0.21) interactions. The Stimulus Frequency X Shape Association X Matching Condition interaction was not significant. Further analysis of the critical Stimulus Frequency X Shape Association interaction, collapsed across Matching Condition (Fig. 3), revealed that when self-related stimuli were presented most frequently during the task, responses were faster to self- compared to friend-related items (*b* =  − 46.05, *SE* = 3.55, *t* = 12.97, *p* < 0.001, *R*^2^ = 0.28). In contrast, when friend-related stimuli were presented most frequently, responses were faster to friend- than to self-related items (*b* = 24.45, *SE* = 3.46, *t* = 7.06, *p* < 0.001, *R*^2^ = 0.21). As indicated by the respective effect sizes, although stimulus prioritization was observed for frequently (vs. infrequently) encountered items, this effect was more pronounced for self-related compared to friend-related stimuli.

#### Accuracy

**Fig. 4:**
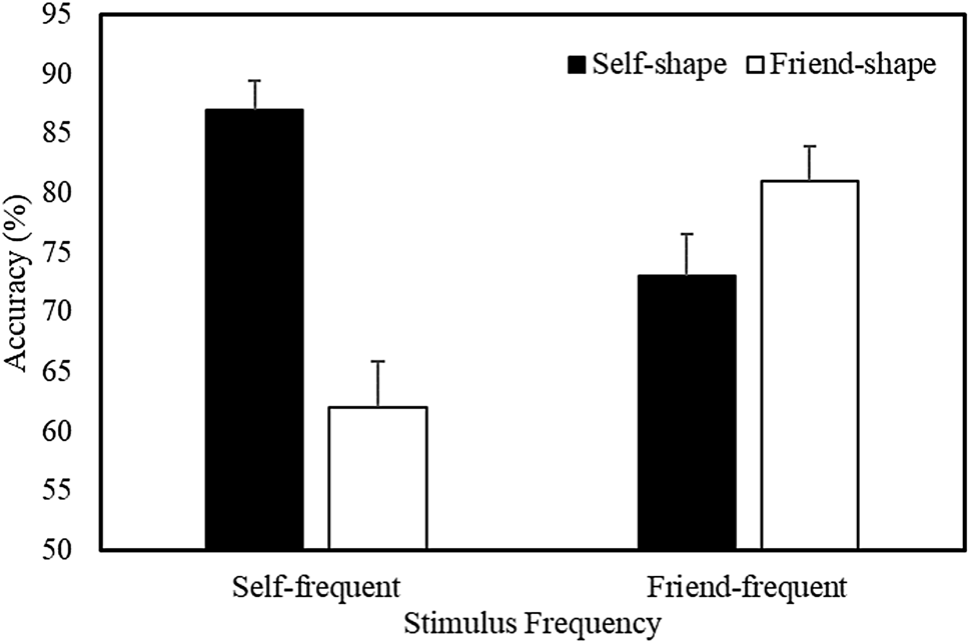
Mean accuracy as a function of Stimulus Frequency and Shape Association (Expt. 2—Confirmatory Context). Error bars represent + 1 SEM

A multilevel logistic regression analysis on the accuracy of responses revealed a main effect of Shape Association (*b* = 0.25, *SE* = 0.03, *z* = 8.34, *p* < 0.001, *R*^2^ = 0.14) and significant Stimulus Frequency X Shape Association *(b* = 0.50, *SE* = 0.03, *z* = 16.30, *p* < 0.001, *R*^2^ = 0.18) and Shape Association X Matching Condition (*b* = 0.14, *SE* = 0.03, *z* = 4.53, *p* < 0.001, *R*^2^ = 0.14) interactions. The Stimulus Frequency X Shape Association X Matching Condition interaction was not significant. Further analysis of the Stimulus Frequency X Shape Association interaction collapsed across Matching Condition (Fig. 4) revealed that, when self-related stimuli were presented most frequently during the task, accuracy was higher for self- compared to friend-related items (*b* = 0.76, *SE* = 0.04, *z* = 17.22, *p* < 0.001, *R*^2^ = 0.23). In contrast, when friend-related stimuli were presented most frequently, accuracy was higher for friend- than self-related items (*b* =  − 0.25, *SE* = 0.04, *z* =  − 5.67, *p* < 0.001, *R*^2^ = 0.14). For the response latencies, prioritization was greater for self-relevant compared to friend-relevant stimuli.

#### Drift diffusion modeling

To explore the processes underpinning task performance, an HDDM analysis was conducted on the data (Wiecki et al., 2013). Three models were estimated for comparison (Table 3). Model 1 (i.e., default model) allowed the drift rate (*v*) to vary as a function of Stimulus Frequency (i.e., self-frequent vs. friend-frequent), Shape Association (i.e., self vs. friend) and Matching Condition (matching vs. nonmatching) and the starting point (*z*) to vary between the response thresholds (i.e., Matching Condition). This model considered whether decision-making was facilitated exclusively by increased information uptake (i.e., stimulus bias), while response-related processes contributed toward a confirmatory matching-bias. The second model was identical to the first, but with the starting point (*z*) varying as a function of Stimulus Frequency to establish if prior beliefs influenced response-related processes (i.e., less decisional evidence was needed for self-related than friend-related matching responses). Finally, in addition to the default (i.e., model 1) parameterization, the third model allowed boundary separation (*a*) to vary as a function of Stimulus Frequency to test whether self- (vs. friend-based) stimulus frequencies altered participants’ decisional style (i.e., conservative vs. liberal). All three models estimated the inter-trial variability for drift rate (*sv*), non-decision time (*st*), and starting point (*sz*).

**Table 3:** Deviance information criterion (DIC) for each model (Expt. 2)

Model	Stimulus frequency	Shape association	Matching condition	DIC
1	*v*	*v*	*z, v*	4160
2	*v, z*	*v*	*z, v*	4156
3	*v, a*	*v*	*z, v*	4154

Note. *v* = drift rate, *z* = starting point, *a* = boundary separation

As can be seen in Table 3, model 3 was the best fit. Analysis of the posterior distributions indicated that task performance was underpinned by differences in processing efficiency (drift rate, *v*), decision-making style (boundary separation, *a*), and the evidential requirements of response generation (starting point, *z*, Table 4). When the self-shape was presented most frequently, there was extremely strong evidence that information uptake (i.e., drift rate) was faster for self-relevant compared to friend-relevant stimuli on both matching (*p*_Bayes_[self > friend] < 0.001) and nonmatching (*p*_Bayes_[self > friend] < 0.001) trials. The opposite effect was observed when the friend-shape was presented most frequently, such that information uptake was faster for friend-related (vs. self-related) items (negative drift rates were first multiplied by − 1) during both matching (*p*_Bayes_[friend > self] = 0.001) and nonmatching (*p*_Bayes_[friend > self] < 0.001) trials. Further comparisons revealed that, during matching trials, information uptake was faster when the frequent stimuli pertained to self rather than to friend (*p*_Bayes_[self-frequent > friend-frequent] < 0.001). This effect was not significant during nonmatching trials (*p*_Bayes_[self-frequent > friend-frequent] = 0.294). In other words, during matching trials, information uptake was fastest when the self-shape (vs. friend-shape) comprised the most frequently encountered stimulus (Table 4).

**Table 4:** Parameter means and the upper (97.5q) and lower (2.5q) quantiles of the best fitting model (Expt. 2)

Diffusion model parameter	Mean	Quantile	
		2.5q	97.5q
a_self-frequent_	1.033	0.938	1.114
a_friend-frequent_	0.995	0.926	1.072
v_self-frequent/matching-self_	2.274	1.906	2.613
v_self-frequent/matching-friend_	0.325	0.062	0.703
v_self-frequent/nonmatching-self_	−1.966	−2.327	−1.602
v_self-frequent/nonmatching-friend_	−0.831	−1.215	−0.44
v_friend-frequent/matching-self_	1.123	0.726	1.505
v_friend-frequent/matching-friend_	1.484	1.314	1.637
v_friend-frequent/nonmatching-self_	−1.136	−1.535	−0.725
v_friend-frequent/nonmatching-friend_	−1.915	−2.287	−1.565
z	0.543	0.527	0.559
t_0_	0.412	0.383	0.445
st	0.365	0.354	0.377
sv	0.087	0.004	0.251
sz	0.406	0.341	0.475

*a* threshold separation, *v* drift rate, *z* starting point, *t*_*0*_ non-decision time, *sv* inter-trial variability in drift rate, *st* inter-trial variability in non-decision time, *sz* inter-trial variability in starting point

Additionally, analysis of the posterior distributions yielded extremely strong evidence that participants adopted a more conservative decisional strategy (i.e., larger *a*) when self-related (vs. friend-related) stimuli appeared more frequently (*p*_Bayes_[self-frequent > friend-frequent] = 0.002). Finally, comparing the starting point value (*z*) with no bias (*z* = 0.50) revealed extremely strong evidence of a response bias in favor of matching (vs. nonmatching) judgments (*p*_Bayes_[bias > 0.50] < 0.001).

## Discussion

Corroborating our prediction, the current results revealed that processing was facilitated for stimuli that appeared most frequently during the shape-label matching task, a prioritization effect that was more pronounced for self- compared to friend-related items (cf. Falbén et al., 2020). This effect, moreover, emerged on both matching and nonmatching trials (cf. Frings & Wentura, 2014; Schäfer et al., 2015, 2016; Sui et al., 2012). As in Experiment 1, decisional bias was underpinned by differences in the rate of information uptake (Golubickis et al., 2018, 2019), but with evidence extracted more rapidly from self-relevant than friend-relevant stimuli. Two other effects were revealed by the HDDM analysis. First, participants were more cautious (i.e., wider threshold separation) when self-relevant rather than friend-relevant stimuli were expected to appear more frequently, suggesting that accuracy was emphasized for the former material. Second, less evidence was needed to generate matching than nonmatching responses, a confirmatory bias that is consistent with previous research exploring the effects of stimulus relevance during shape-label matching tasks (Golubickis et al., 2017, 2020).

A key feature of Experiments 1 and 2 was that prior beliefs were predictive with respect to the forthcoming experimental trials (De Loof et al., 2016; Dunovan et al., 2014; Falbén et al., 2020). That is, having been told to expect an equivalent number of self-related and friend-related items or that the highest frequency of shapes would comprise either self-related or friend-related stimuli, this was indeed the case. A natural question to ask, therefore, is what would happen in a dis-confirmatory task context in which the supposedly minority stimuli are encountered most frequently? In their object-ownership task, under just such conditions, Falbén et al., (2020; Expt. 3) demonstrated that inaccurate priors were overridden in accordance with the stimuli that predominated during the task. That is, frequent (vs. infrequent) stimuli were prioritized during decisional processing. This indicates that participants optimized a probabilistic representation of the immediate task environment, triggering stimulus enhancement for the most prevalent items (Bar, 2007; Chater & Oaksford, 2008; O’Callaghan et al., 2017; Otten et al., 2017). But would comparable effects emerge in a shape-label matching task? Given the results of Experiment 2 (i.e., personal relevance augments the effects of stimulus frequency), we hypothesized that frequently (vs. infrequently) encountered stimuli would be prioritized in a dis-confirmatory task setting. However, this effect would be larger for self-related compared to friend-related items (cf. Falbén et al., 2020).

Of additional theoretical interest in dis-confirmatory task contexts are the temporal characteristics of stimulus prioritization. If participants initially adopt an expectancy based on the experimental instructions (i.e., prior belief), but later update this belief in accordance with the actual frequency with which stimuli are encountered (i.e., posterior belief), then performance may be influenced by the strength of the initial expectancy. That is, strong (vs. weak) expectancies may be less susceptible to modification. Given the demonstration in Experiment 2 that stimulus prioritization was greater for self- than friend-related items, this suggests that self- and friend-related expectancies may differ in stability/persistence in the face of disconfirmation (Wang et al., 2016). Acknowledging this possibility, in our next experiment, we explored the dynamic effects of expectancy disconfirmation during the shape-label matching task.

## Experiment 3: dis-confirmatory context

### Method

#### Participants and design

Twenty-five undergraduates (18 female, *M*_age_ = 20.4, *SD* = 2.75) took part in the experiment. All participants had normal or corrected-to-normal visual acuity. Informed consent was obtained from participants prior to the commencement of the experiment and the protocol was reviewed and approved by the Ethics Committee at the School of Psychology, University of Aberdeen. The experiment had 2 (Stimulus Frequency: self-frequent or friend-frequent) X 2 (Shape Association: self or friend) X 2 (Matching Condition: matching or nonmatching) X Trial Number repeated-measures design.

### Stimulus materials and procedure

Participants arrived at the laboratory individually, were greeted by an experimenter, seated at a desktop computer, and informed that the study comprised a matching task featuring geometric shapes that represented them (i.e., self) and their best friend (Sui et al., 2012). The task closely followed Experiment 2, but with a different manipulation of stimulus-related frequencies. Across two blocks of trials, following Falbén et al., (2020), although participants were told to expect a higher frequency of either the self-shape (i.e., 75% self & 25% friend) or the friend-shape (i.e., 75% friend & 25% self) to be presented during the task, in reality, the opposite was the case (i.e., self-frequent: 25% self-shape & 75% friend-shape; friend-frequent: 25% friend-shape & 75% self-shape). The order of the experimental blocks was counterbalanced across participants and each block comprised 200 trials. The self-frequent (i.e., friend-expectancy) block consisted of 100 matching and 100 nonmatching trials. Of these, 150 trials comprised self-related shape-label pairings, with 75 matching and 75 nonmatching trials. The remaining 50 trials comprised friend-related shape-label pairings, with 25 matching and 25 nonmatching trials. The friend-frequent (i.e., self-expectancy) block had an identical trial structure, but with friend-related shape-label pairings presented most frequently. The order of the trials was randomized in each block. On completion of the task, participants were debriefed, thanked, and dismissed.

## Results

### Response time

Responses faster than 200 ms were excluded from the analysis (Sui et al., 2012), eliminating less than 2% of the overall trials. A multilevel model analysis was used to examine the correct RT and accuracy data (see Table S3 in the Supplementary Material for a listing of all the treatment means). Stimulus Frequency, Shape Association, and Matching Condition were treated as categorical fixed effects, Trial Number as a continuous variable, and participants as a crossed random effect. This yielded main effects of Stimulus Frequency (*b* = 11.11, *SE* = 4.66, *t* = 2.38, *p* = 0.01, *R*^2^ = 0.18), Shape Association (*b* =  − 13.74, *SE* = 4.66, *t* =  − 2.95, *p* = 0.01, *R*^2^ = 0.19), and Matching Condition (*b* =  − 20.50, *SE* = 4.67, *t* =  − 4.39, *p* < 0.001, *R*^2^ = 0.20), and significant Stimulus Frequency X Shape Association (*b* = 12.39, *SE* = 4.66, *t* = 2.66, *p* = 0.01, *R*^2^ = 0.20) and Stimulus Frequency X Shape Association X Trial Number (*b* = 0.12, *SE* = 0.04, *t* = 3.02, *p* = 0.002, *R*^2^ = 0.20) interactions. The Stimulus Frequency X Shape Association X Matching Condition interaction was not significant.

**Fig. 5:**
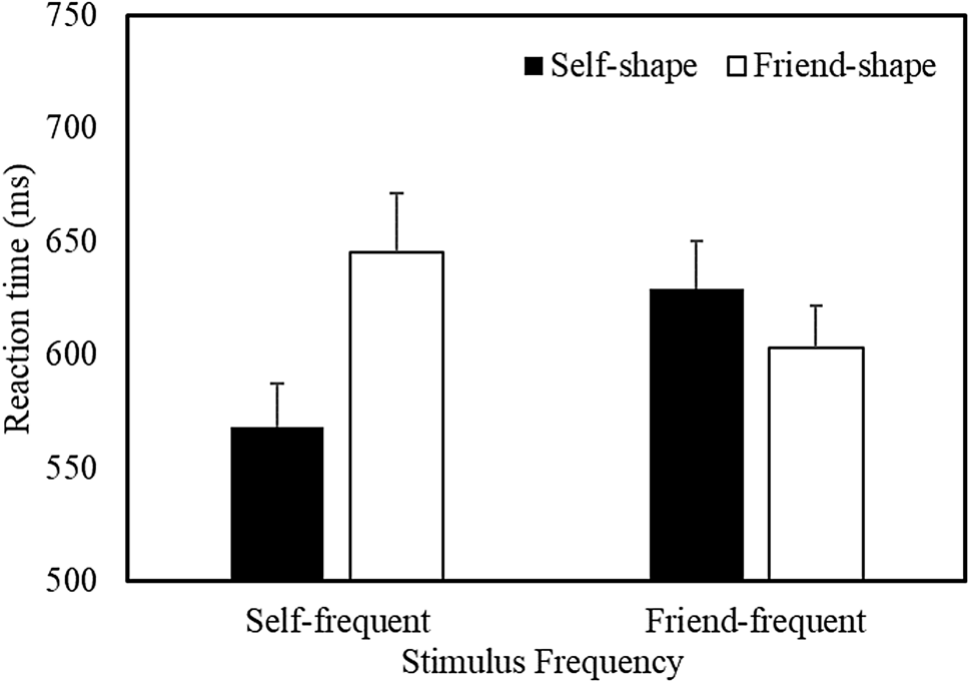
Mean response time as a function of Stimulus Frequency and Shape Association (Expt. 3—Dis-confirmatory Context). Error bars represent + 1 SEM

Further inspection of the critical Stimulus Frequency X Shape association interaction collapsed across Matching Condition (Fig. 5) revealed that, when self-related stimuli were presented most frequently during the task, responses were faster to self-than to friend-related items (*b* =  − 38.08, *SE* = 3.41, *t* =  − 11.17, *p* < 0.001, *R*^2^ = 0.25). In contrast, when friend-related stimuli were presented most frequently, responses were faster to friend- than to self-related items (*b* = 12.12, *SE* = 3.23, *t* = 3.75, *p* = 0.01, *R*^2^ = 0.22). As revealed by the respective effect sizes, stimulus prioritization was most pronounced when self-related (vs. friend-related) items predominated during the task.

To explore the dynamic character of these effects, separate Shape Association X Trial Number analysis was undertaken for each of the Stimulus Frequency conditions. When the self-related shape predominated, only a main effect of Shape Association (*b* =  − 29.68, *SE* = 6.75, *t* =  − 4.39, *p* < 0.001, *R*^2^ = 0.25) was observed. When the friend-related shape was presented most frequently during the task, a main effect of Trial Number (*b* =  − 0.11, *SE* = 0.05, *t* = 1.20, *p* = 0.048, *R*^2^ = 0.22) and a significant Shape Association X Trial Number interaction (*b* = 0.13, *SE* = 0.05, *t* = 2.33, *p* = 0.02, *R*^2^ = 0.22) emerged. Further inspection of the interaction indicated that, when friend-related stimuli appeared more frequently, whereas response times toward these items decreased over the course of the experiment (*b* =  − 0.24, *SE* = 0.05, *t* =  − 4.25, *p* < 0.001, *R*^2^ = 0.23), there was no significant change in response times toward self-related stimuli.

#### Accuracy

A multilevel logistic regression analysis on the accuracy of responses revealed a main effect of Shape Association (*b* = 0.34, *SE* = 0.058, *z* = 5.87, *p* < 0.001, *R*^2^ = 0.09) and significant Stimulus Frequency X Shape Association *(b* =  − 0.18, *SE* = 0.06, *z* =  − 3.15, *p* = 0.001, *R*^2^ = 0.12), Shape Association X Matching Condition (*b* = 0.24, *SE* = 0.06, *z* = 4.16, *p* < 0.001, *R*^2^ = 0.10), Stimulus Frequency X Shape Association X Trial Number (*b* =  − 0.001. *SE* < 0.001, *z* =  − 3.18, *p* = 0.001, *R*^2^ = 0.12), Shape Association X Matching Condition X Trial Number (*b* =  − 0.001. SE < 0.001, *z* =  − 2.18, *p* = 0.03, *R*^2^ = 0.10), and Stimulus Frequency X Shape Association X Matching Condition X Trial Number (*b* = 0.001, *SE* < 0.001, *z* = 2.05, *p* = 0.04, *R*^2^ = 0.13) interactions.

**Fig. 6:**
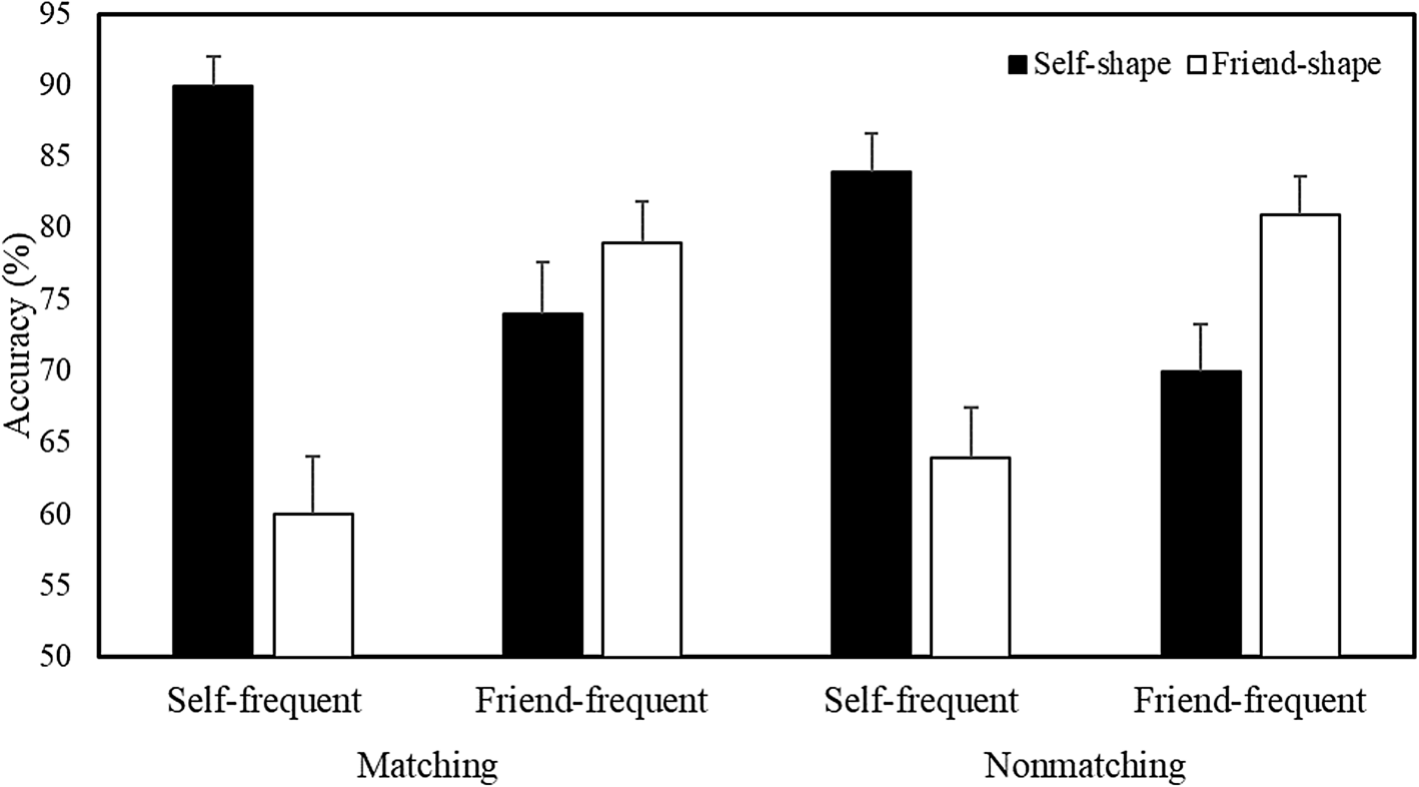
Mean accuracy as a function of Stimulus Frequency, Shape Association, and Matching Condition (Expt. 3—Dis-confirmatory Context). Error bars represent + 1 SEM

To explore further the four-way interaction, separate Stimulus Frequency X Shape Association X Trial Number analyses were undertaken for each Matching Condition (Fig. 6). On matching trials, this yielded a main effect of Shape Association (*b* = 0.60, *SE* = 0.08, *z* = 7.25, *p* < 0.001, *R*^2^ = 0.15), and significant Shape Association X Trial Number (*b* =  − 0.001, *SE* = 0.007, *z* =  − 2.52, *p* = 0.01, *R*^2^ = 0.15) and Stimulus Frequency X Shape Association (*b* =  − 0.30, *SE* = 0.08, *z* =  − 3.58, *p* < 0.001, *R*^2^ = 0.17) interactions. When the self-shape was presented most frequently during the task, accuracy was higher for self-related compared to friend-related stimuli (*b* = 0.74, *SE* = 0.06, *z* = 12.86, *p* < 0.001, *R*^2^ = 0.19). Conversely, when the friend-shape comprised the most frequent stimulus, no significant difference in accuracy was observed. Thus, stimulus prioritization only emerged when self-related shapes predominated.

On nonmatching trials, the analysis yielded a significant Stimulus Frequency X Shape Association X Trial Number (*b* =  − 0.002, *SE* < 0.001, *z* =  − 3.35, *p* < 0.001, *R*^2^ = 0.14) interaction. As such, separate Shape Association X Trial Number analyses were undertaken for each of the Stimulus Frequency conditions. When the self-shape was presented most frequently during the task, the analysis revealed a Shape Association X Trial Number (*b* = 0.003, *SE* = 0.001, *z* = 3.15, *p* = 0.001, *R*^2^ = 0.20) interaction. Whereas accuracy for the friend-shape decreased over time (*b* =  − 0.004, *SE* = 0.001, *z* =  − 2.30, *p* = 0.02, *R*^2^ = 0.11), no significant difference was observed for the self-shape. When the friend-shape comprised the most frequent stimulus, the analysis also revealed a Shape Association X Trial Number (*b* =  − 0.002, *SE* = 0.001, *z* =  − 2.03, *p* = 0.04, *R*^2^ = 0.11) interaction. Whereas accuracy on self-related trials decreased over time (*b* =  − 0.003, *SE* = 0.001, *z* =  − 1.95, *p* = 0.05, *R*^2^ = 0.09), no significant difference was observed on friend-related trials.

#### Drift diffusion modeling

As previously, an HDDM analysis was performed on the data. Extending Experiment 2, a dynamic-bias approach was adopted to model the data. Of theoretical interest was exploring the time course of decisional processing in a task context in which stimulus-related expectancies pertaining to the self and a friend were disconfirmed. Previous research on this topic has demonstrated that expectancy violation influences the parameters of decision-making, suggesting that response and stimulus biases are sensitive both to prior information (i.e., expectations provided to participants) and trial-by-trial sensory experiences (Falbén et al., 2020). What is not yet known, however, is whether these decisional processes are sensitive to disconfirmation as it unfolds over the course of the task. As such, here we considered the temporal dynamics of decisional bias during shape-label matching. To do so, Trial Number was regressed as a continuous variable for each parameter estimate. Following Experiment 2, for model comparison, three model parameterizations were conducted. Model 3 was identified as the best fitting (Table 5).

**Table 5:** Deviance information criterion (DIC) for each model (Expt. 3)

Model	Stimulus frequency	Shape association	Matching condition	Trial number	DIC
1	*v*	*v*	*z, v*	*z, v, a*	4851
2	*v, z*	*v*	*z, v*	*z, v, a*	4849
3	*v, a*	*v*	*z, v*	*z, v, a*	4842

*v* drift rate, *z* starting point, *a* boundary separation

Analysis of the best fitting model revealed that decision-making was underpinned by variation in information uptake (drift rate, *v*), decision-making style (boundary separation, *a*), and the evidential requirements of response generation (starting point, *z*, Table 6). In addition, dynamic changes in some of these biases were also observed. First, in the friend-frequent condition (i.e., self-shapes were expected to appear more frequently, but did not), there was extremely strong evidence that information uptake (i.e., drift rate) was faster for self-related compared to friend-related stimuli (negative drift rates were first multiplied by − 1) during matching trials (*p*_Bayes_[self > friend] < 0.001). In addition, there was extremely strong evidence for an increase in the rate of information uptake for friend-related stimuli during matching trials over the course of the task (*p*_Bayes_[friend: Trial Number] < 0.001), and extremely strong evidence for a decrease in the rate of information uptake for these items during nonmatching trials (*p*_Bayes_[friend: Trial Number] = 0.005). In the self-frequent condition (i.e., friend-shapes were expected to appear more frequently, but did not), there was extremely strong evidence that the rate of information uptake was faster for self-related than friend-related stimuli on both matching (*p*_Bayes_[self > friend] < 0.001) and nonmatching (*p*_Bayes_[self > friend] = 0.04) trials. In addition, during nonmatching trials, the rate of information uptake for friend-related stimuli increased over time (*p*_Bayes_[friend: Trial Number] = 0.02).

**Table 6:** Parameter means and the upper (97.5q) and lower (2.5q) quantiles of the best fitting model (Expt. 3)

Diffusion model parameter	Mean	Quantile	
		2.5q	97.5q
a_friend-frequent_	1.046	0.898	1.252
a_self-frequent_	0.96	0.892	1.032
v_friend-frequent/matching-self_	1.987	1.317	2.598
v_friend-frequent/matching-friend_	0.954	0.477	1.402
v_friend-frequent/nonmatching-self_	− 1.632	− 2.302	− 0.959
v_friend-frequent/nonmatching-friend_	− 1.515	− 2.032	− 1.041
v_self-frequent/matching-self_	2.521	1.929	3.114
v_self-frequent/matching-friend_	0.672	0.477	0.882
v_self-frequent/nonmatching-self_	− 1.857	− 2.397	− 1.262
v_self-frequent/nonmatching-friend_	− 1.346	− 1.857	− 0.754
z	0.505	0.486	0.425
t_0_	0.415	0.404	0.42
st	0.44	0.422	0.448
sv	0.102	0.002	0.233
sz	0.787	0.677	0.849
Regression coefficients (trial number)			
a_friend-frequent_	− 4.44E-04	− 0.001	− 4.62E-05
a_self-frequent_	2.65E-05	− 4.11E-04	4.98E-04
v_friend-frequent/matching-self_	− 0.002	− 0.006	0.001
v_friend-frequent/matching-friend_	0.005	0.002	0.007
v_friend-frequent/nonmatching-self_	0.002	− 0.001	0.003
v_friend-frequent/nonmatching-friend_	− 0.003	− 0.005	− 0.001
v_self-frequent/matching-self_	− 0.001	− 0.002	0.004
v_self-frequent/matching-friend_	− 0.001	− 0.003	0.001
v_self-frequent/nonmatching-self_	− 0.001	− 0.003	0.001
v_self-frequent/nonmatching-friend_	0.005	0.001	0.009
z	− 5.80E-05	− 2.10E-04	1.21E-04

*a* boundary separation, *v* drift rate, *z* starting point, *t*_*0*_ non-decision time, *sv* inter-trial variability in drift rate, *st* inter-trial variability in non-decision time, *sz* inter-trial variability in starting point

Second, there was strong evidence that participants adopted a more conservative decisional strategy (i.e., larger *a*) when self-shapes were expected to be more frequent (i.e., friend-shapes predominated) than vice versa (*p*_Bayes_[friend-frequent > self-frequent] = 0.017). The trial-by-trial analysis revealed that decisional caution reduced over the course of the task when self-shapes were expected to be the most frequent but friend-shapes predominated (*p*_Bayes_[friend-frequent: Trial Number] = 0.01). Finally, comparison of the observed starting value (*z*) with no bias (*z* = 0.50) indicated no evidence of a response bias (*p*_Bayes_[bias > 0.50] = 0.35).

## Discussion

The current findings extend the effects reported in Experiments 1 and 2. On both matching and nonmatching trials, whether participants expected self-shapes or friend-shapes to comprise the most frequently presented items, responses were speeded for the shapes that actually predominated during the task. As previously, however, stimulus prioritization was greater for self- compared to friend-related stimuli. Regarding the accuracy of performance, self-prioritization emerged when self-shapes predominated during matching trials. Across the course of the shape-label matching task, self- and friend-related expectancies also yielded some interesting dynamic differences. Specifically, when participants expected the self-shape to be presented most frequently, but it was the friend-shape that actually predominated, response latencies to the friend-shape decreased over time and no difference was observed for the self-shape. In contrast, when participants expected the friend-shape to be presented most frequently, but the self-shape predominated, response latencies were faster to the self-shape (vs. friend-shape) throughout the task. This suggests that, compared to the friend-related expectancy, prior beliefs about the self were less malleable (Wang et al., 2016).

Corroborating Experiment 2, decisional bias was underpinned by a stimulus bias, with information uptake faster for self- compared to friend-relevant stimuli. Interestingly, however, dynamic changes in stimulus processing were also observed, but only for friend-related items. Notably, when the friend-related stimulus was presented most frequently, the rate of information uptake for this item increased over time. Similarly, when the self-shape predominated, dynamic changes in information uptake were observed only for the friend-related stimulus. This indicates that, compared to friend-relevant material, the processing of self-relevant stimuli is less susceptible to modification in a task context in which prior beliefs regarding the frequency of stimulus presentation are disconfirmed. As in Experiment 2, the HDDM analysis also revealed differences in decision-making style, such that participants were more cautious (i.e., wider threshold separation) when self-related (vs. friend-related) stimuli were expected to appear more frequently, an effect that diminished over time.

### Additional analysis

To explore the effects of stimulus frequency across confirmatory and dis-confirmatory task contexts, an additional analysis was undertaken by combining the data from Experiments 2 and 3. A multilevel model analysis was used to examine the correct RT and accuracy data. Task Context (confirmatory or dis-confirmatory), Stimulus Frequency (self-frequent or friend-frequent), Shape Association (self or friend), and Matching Condition (matching or nonmatching) were treated as categorical fixed effects, and participants as a crossed random effect.

### Response time

The analysis yielded main effects of Shape Association (*b* = -10.75, *SE* = 1.71, *t* =  − 6.27, *p* < 0.001, *R*^2^ = 0.19), and Matching Condition (*b* =  − 24.14, *SE* = 1.72, *t* =  − 14.06*, p* < 0.001, *R*^2^ = 0.21), and significant Task Context X Stimulus Frequency (*b* = 6.70, *SE* = 1.72, *t* = 3.90, *p* < 0.001, *R*^2^ = 0.19), Stimulus Frequency X Shape Association (*b* =  − 29.90, *SE* = 1.71, *t* =  − 17.43, *p* < 0.001, *R*^2^ = 0.21), Shape Association X Matching Condition (*b* =  − 7.92, *SE* = 1.71, *t* =  − 4.62, *p* < 0.001, *R*^2^ = 0.21), Task Context X Stimulus Frequency X Shape Association (*b* = -5.22, *SE* = 1.71, *t* =  − 3.04, *p* = 0.002, *R*^2^ = 0.21), and Stimulus Frequency X Shape Association X Matching Condition (*b* = 3.53, *SE* = 1.71,* t* = 2.06, *p* = 0.04, *R*^2^ = 0.23) interactions. The Task Context X Stimulus Frequency X Shape Association X Matching Condition interaction was not significant.

**Fig. 7:**
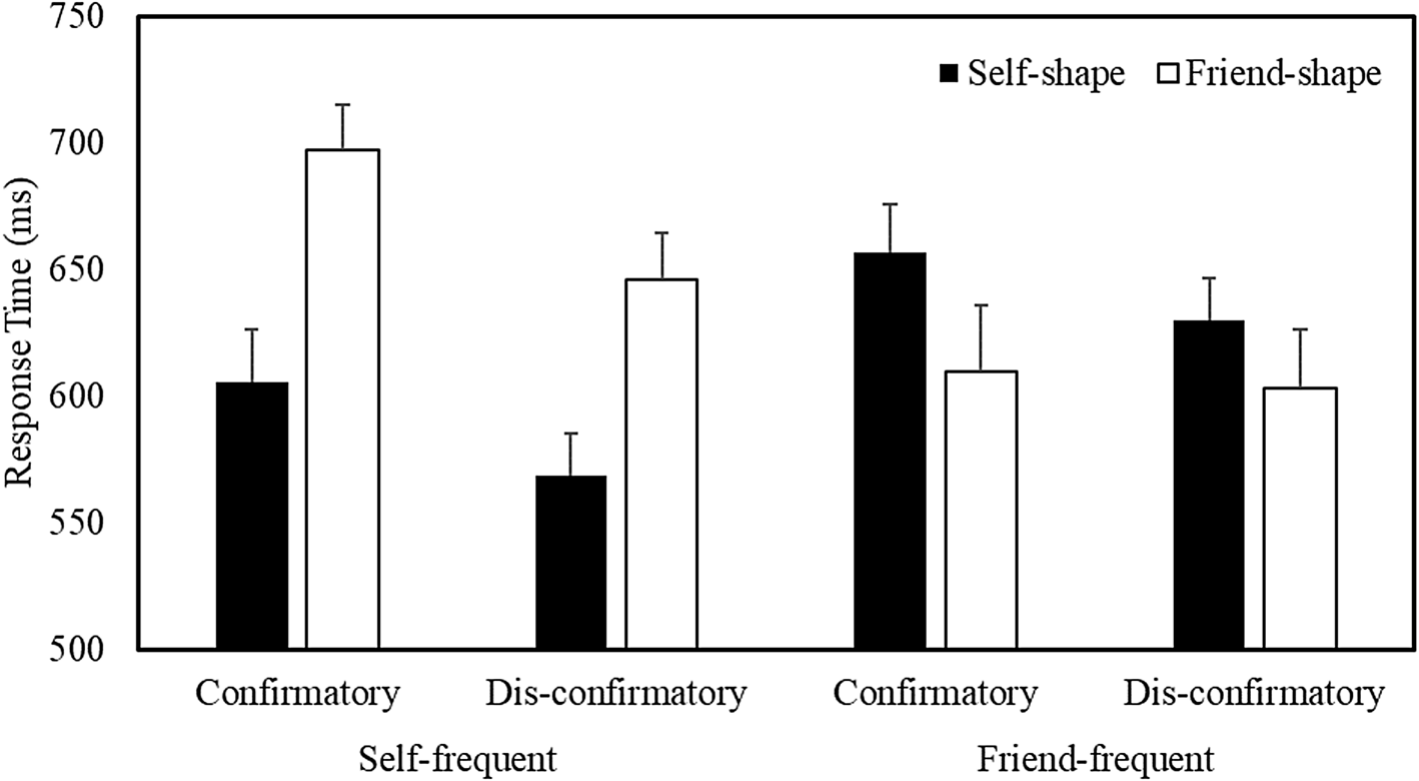
Mean response time as a function of Task Context, Stimulus Frequency, and Shape Association (Expts. 2 & 3 combined). Error bars represent + 1 SEM

To further explore the critical Task Context X Stimulus Frequency X Shape Association interaction, separate Task Context X Shape Association analyses were conducted for each Stimulus Frequency collapsed across Matching Condition (Fig. 7). When self-related stimuli were presented most frequently, the analysis yielded a main effect of Shape Association, such that responses were faster to self- compared to friend-related items (*b* =  − 24.40, *SE* = 2.03, *t* =  − 12.04, *p* < 0.001, *R*^2^ = 0.27). When friend-related items comprised the most frequently encountered stimuli, the analysis yielded a main effect of Shape Association, with responses faster to friend- than self-related items (*b* = 11.81, *SE* = 2.06, *t* = 5.73, *p* < 0.001, *R*^2^ = 0.21).

### Accuracy

**Fig. 8:**
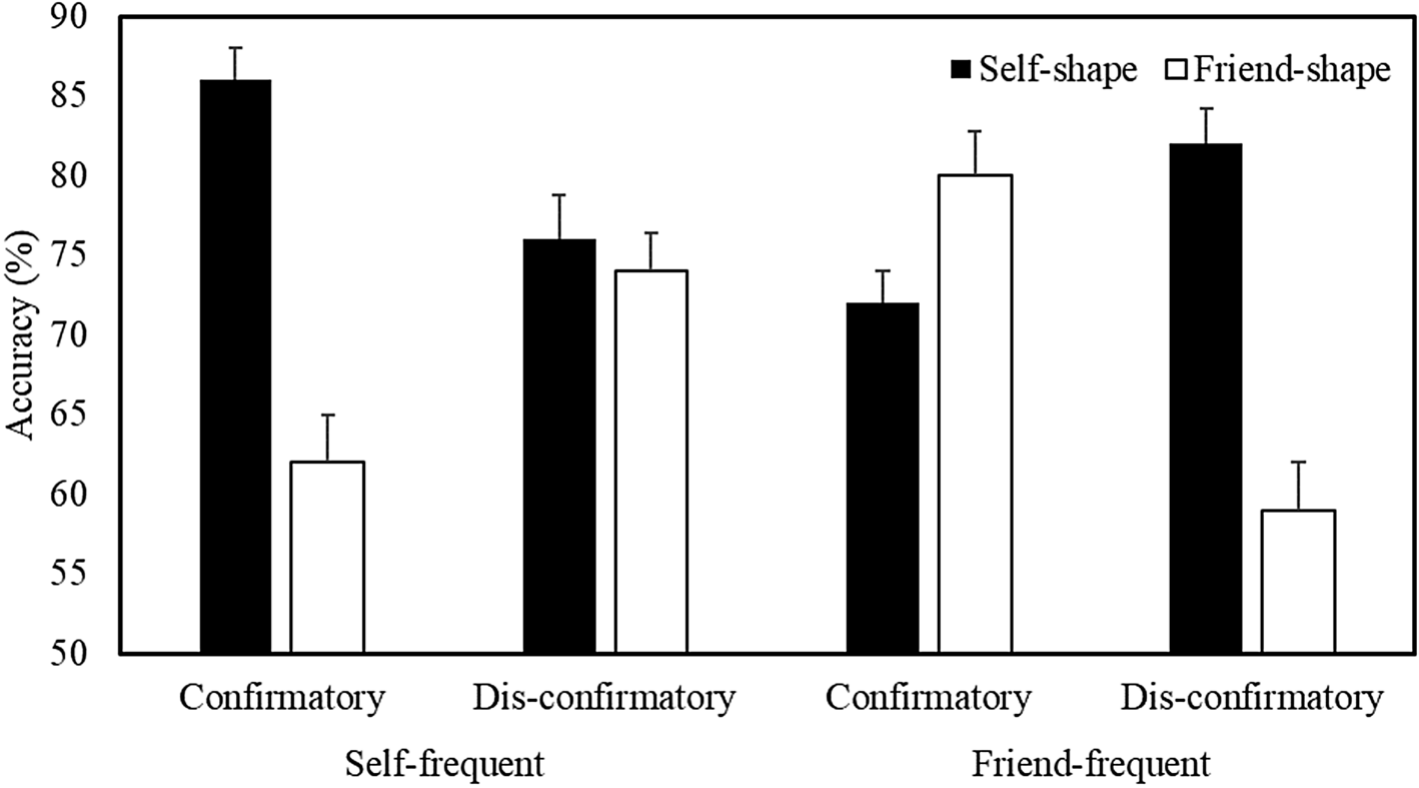
Mean accuracy as a function of Task Context, Stimulus Frequency, and Shape Association (Expts. 2 & 3 combined). Error bars represent + 1 SEM

A multilevel logistic regression analysis on the accuracy of responses revealed a main effect of Shape Association (*b* = 0.26, *SE* = 0.02, *z* = 12.68, *p* < 0.001, *R*^2^ = 0.12), and significant Stimulus Frequency X Shape Association (*b* = 0.42, *SE* = 0.02, *z* = 20.04, *p* < 0.001, *R*^2^ = 0.15), Shape Association X Matching Condition (*b* = 0.13, *SE* = 0.02, *z* = 6.51, *p* < 0.001, *R*^2^ = 0.13), and Task Context X Stimulus Frequency X Shape Association (*b* = 0.08, *SE* = 0.02, *z* = 3.81, *p* = 0.001, *R*^2^ = 0.16) interactions. The Task Context X Stimulus Frequency X Shape Association X Matching Condition interaction was not significant. To explore further the critical three-way interaction, separate Task Context X Shape Association analyses were conducted for each Stimulus Frequency collapsed across Matching Condition (Fig. 8).

When self-related stimuli appeared most frequently, the analysis yielded a main effect of Shape Association (*b* = 0.69, *SE* = 0.03, *z* = 23.12, *p* < 0.001, *R*^2^ = 0.21) and a significant Task Context X Shape Association (*b* = 0.06, *SE* = 0.03, *z* = 2.22, *p* = 0.02, *R*^2^ = 0.21) interaction. Participants responded more accurately to self- compared to friend-related stimuli in both a confirmatory (*b* = 0.75, *SE* = 0.04, *z* = 17.22, *p* < 0.001, *R*^2^ = 0.23) and dis-confirmatory (*b* = 0.62, *SE* = 0.04, *z* = 15.42, *p* < 0.001, *R*^2^ = 0.18) task context, but this effect was most pronounced in the former setting. When friend-related stimuli were encountered most frequently during the task, the analysis again revealed a main effect of Shape Association (*b* =  − 0.15, *SE* = 0.03, *z* =  − 5.13, *p* < 0.001, *R*^2^ = 0.10) and a significant Task Context X Shape Association (*b* =  − 0.09, *SE* = 0.03, *z* =  − 3.10, *p* = 0.002, *R*^2^ = 0.11) interaction. Whereas participants responded more accurately to friend- compared to self-related stimuli in a confirmatory task context (*b* =  − 0.24, *SE* = 0.04, *z* =  − 5.67, *p* < 0.001, *R*^2^ = 0.14), this effect was not significant in a dis-confirmatory setting.

## Discussion

The combined analyses revealed that, during both matching and nonmatching trials, stimulus prioritization was driven by the frequency with which items were presented in combination with the personal relevance of the material. Specifically, across both confirmatory and dis-confirmatory task contexts, frequently (vs. infrequently) presented items were prioritized during decisional processing when they were associated with the self. When friend-related items predominated, a comparable effect emerged only in a confirmatory task context. Thus, while item frequency triggers stimulus prioritization, self-relevance provides an extra benefit during decisional processing (Humphreys & Sui, 2016; Reuther & Chakravarthi, 2017; Sui & Humphreys, 2015, 2017).

### General discussion

Numerous studies have revealed the benefits of self-relevance on decision-making (Humphreys & Sui, 2016; Sui & Humphreys, 2015, 2017). Compared to stimuli associated with other people, those paired with the self are prioritized during decisional processing. Developing work on this topic, here we demonstrated that prioritization was influenced by the personal significance of stimuli in combination with the frequency in which they were encountered. Specifically, when self- and friend-related stimuli were equally likely to be encountered during a shape-label matching task, a self-prioritization effect emerged (Expt. 1). In addition, in confirmatory (Expt. 2) and dis-confirmatory (Expt. 3) task contexts, frequently (vs. infrequently) encountered items were prioritized both for the self and a friend (cf. Sui et al., 2014). Crucially, however, stimulus prioritization was greater for self- compared to friend-related items. Underpinning the facilitated responding in each of the reported experiments was variation in the rate of information uptake during decision-making (Golubickis et al., 2017, 2020), with evidence extracted more rapidly from self-related compared to friend-related stimuli.

Despite the adoption of a broadly similar task context, the current results failed to corroborate prior work exploring the effects of item frequency on the emergence of self-prioritization. Using a sequential version of the shape-label matching task, Sui et al., (2014) reported that self-bias was largely unaffected by the frequency of stimulus presentation, with self-prioritization emerging even when self-relevant stimuli were presented only occasionally. Moreover, although a stimulus-prioritization effect was observed when mother-related items predominated during the task, this benefit was contingent upon stranger being the target of comparison. When mother-related items comprised the predominant stimuli and self-related items appeared less frequently, task performance was comparable. Crucially, however, Sui et al. (2014) provided no prior information to participants regarding the frequency of stimulus presentation and employed two targets of self-other comparison (i.e., mother & stranger), an additional factor that may have contributed to the reported effects. Given the current findings (see also Falbén et al., 2020), it appears that item prioritization may be more pliable when stimulus-related expectations are furnished before the matching task commences.

While an extensive literature has demonstrated self-prioritization during shape-label matching trials (Frings & Wentura, 2014; Reuther & Chakravarthi, 2017; Schäfer et al., 2015, 2016; Sui et al., 2012), comparable effects on nonmatching trials have been less frequent with stimulus enhancement sometimes even going in the wrong direction (Payne et al., 2017; Stolte et al., 2017). Accordingly, in the current investigation, no hypotheses were made regarding nonmatching trials. Interestingly, however, significant effects were observed. As revealed by the combined analysis, stimulus prioritization emerged on both matching and nonmatching trials for frequently (vs. infrequently) encountered items. What this suggests is that prior beliefs sensitized participants to the actual presentation of self- and friend-related items during the task, ultimately triggering stimulus prioritization regardless of whether matching or nonmatching responses were required. In other words, the relative contribution of top–down (i.e., prior beliefs) and bottom–up attentional operations during decisional processing varied as a function of the task context, such that properties of the stimuli (e.g., frequency of presentation, personal relevance) dominated processing when the to-be-judged items were made salient (Humphreys & Sui, 2016; Sui & Humphreys, 2015, 2017). Although it is commonplace for researchers to exclude nonmatching trials from statistical analysis (Hu et al., 2020; Schäfer et al., in press; Sel et al., 2019), the current results caution against this approach. As a function of the prevailing task context, there would appear to be conditions under which self-prioritization emerges regardless of trial type (Janczyk et al., 2019; Moradi et al., 2015; Sui et al., 2014). Future research should therefore clarify exactly when and how self-relevance influences performance during nonmatching shape-label trials.

### Triggering self-prioritization

In a world of daunting complexity, mechanisms are needed to direct attention to task-relevant stimuli (e.g., a red light at the intersection) while downplaying the appeal of distracting objects (e.g., a colleague on the sidewalk). To realize these objectives, models of selective attention posit the interplay between two processes, voluntary (i.e., goal-directed) and automatic (i.e., stimulus-driven) attentional control (e.g., Corbetta & Shulman, 2001; Itti & Koch, 2001; Theeuwes, 2010), such that information processing is driven by a combination of current goals and priorities and the physical properties of the stimulus environment (e.g., salience of objects). Within this general framework, Humphreys and Sui (2015) have argued that, much like the effects of perceptual salience, self-prioritization reflects the operation of an automatic attentional process that alters the potency of personally meaningful material (Sui & Humphreys, 2016, 2017; Sui & Rotshtein, 2019). According to this account, self-prioritization is a pivotal, stimulus-driven property of mental life. As Sui and Rotshtein (2019, p. 151) have put it, “…self-relevance acts like a gold thread to facilitate processing of information.”

Although certain exemplars of self-relevant stimuli—including one’s face or forename—may be prioritized automatically (e.g., Alexopoulos et al., 2012; Bargh, 1982; Keyes & Brady, 2010; Moray, 1959; but see Alzueta et al., 2019), effects of this kind probably originate in the familiarity of the items. Indeed, when arbitrary objects comprise the stimuli of interest, evidence to support the mandatory prioritization of self-relevant material is conspicuously lacking (Caughey et al., 2021). Instead, stimulus prioritization necessitates the operation of goal-directed processing (Posner & Petersen, 1990). For example, using breaking continuous flash suppression (b-CFS) to explore the ease with which Gabor patches enter consciousness, Stein et al. (2016) found no advantage for self-relevant stimuli (cf. Macrae et al., 2017). Interesting, however, in a prior Gabor-label perceptual-matching task, a standard self-prioritization effect was observed. In much the same way, Siebold et al. (2015) reported no evidence that self-relevance enhanced stimulus (i.e., lines) detection in a rapid visual search paradigm, although a self-prioritization effect was observed in an earlier line-label matching task. Outside the realm of explicit shape-label matching tasks, findings such as these challenge the contention that self-prioritization is an obligatory stimulus-driven phenomenon. Absent task instructions that direct attention to the self-relevance (or otherwise) of stimuli, prioritization reliably fails to arise (Caughey et al., 2021; Constable et al., 2019a, 2019b; Falbén et al., 2019).

Falling short of compulsory, the benefits of self-relevance are nevertheless considerable (Humphreys & Sui, 2016; Sui & Humphreys, 2015, 2017). Here, we showed that stimulus prioritization was impacted by the frequency with which items were encountered in tandem with the personal relevance of the to-be-judged items. Crucially, although stimulus prioritization was observed for frequently presented items whether they pertained to the self or a friend, this effect was augmented by the relevance of the material, such that processing gains were larger for stimuli that related to the self (i.e., Expts. 2 & 3). In other words, consistent with the SAN model (Humphreys & Sui, 2016; Sui & Humphreys, 2015, 2017), the benefits of self-relevance supplemented the effects of stimulus frequency. Contrasting Falbén et al. (2020), the absence of powerful response-related expectancies in shape-label matching tasks likely triggered these differences in stimulus prioritization. Indeed, reflecting the benefit that personal relevance exerts during decisional processing, a self-prioritization effect emerged even when prior instructions indicated that equivalent numbers of self- and friend-related stimuli would be encountered during the task (i.e., Expt. 1). This indicates that probabilistic manipulations were accompanied by a default bias toward self-relevant material during decision-making (Humphreys & Sui, 2015; Sui & Humphreys 2015, 2017 ).

Further exploring the character of stimulus prioritization, a dynamic analysis yielded evidence for the stability of self-bias. The results of Experiment 3 revealed that prior stimulus-based expectations were impacted by the actual frequency of stimulus presentation, irrespective of the target with which the items had been associated (cf. Sui et al., 2014). Nevertheless, associations formed in relation to the self (vs. friend) induced a more persistent bias, such that beliefs about the self were less susceptible to updating on the basis of trial-by-trial information. That is, stimulus-related beliefs about the self (vs. friend) were resistant to modification in the face of disconfirmation. Relatedly, using a relearning paradigm, Wang et al. (2016) demonstrated participants’ difficulties overcoming prior self-shape (vs. friend-shape) associations when forming new target-shape relations. Although speculative, this property of self-bias would clearly facilitate the maintenance of stable beliefs about the self, an essential component of social–cognitive functioning (Greenwald, 1980; Markus, 1977). An intriguing possibility is that, compared to beliefs pertaining to other persons, prior expectations about the self are weighted more heavily, thus less sensitive to dis-confirmatory sensory inputs. A useful direction for future research will therefore be to explicate how a range of factors—including goals, needs, and preferences—influence the processes (top–down & bottom–up) that underpin the emergence and stability of self-prioritization across a range of task contexts.

### Pathways to stimulus prioritization

Dominant accounts of self-prioritization assert that stimulus relevance moderates the efficiency of visual processing (Humphreys & Sui, 2016; Sui & Humphreys, 2015, 2017). Specifically, through increased social salience, self-relevance (vs. other relevance) facilitates the perceptual appraisal of stimuli. Evidence supporting this viewpoint is limited, however, and derived primarily from work exploring the neural correlates of self-prioritization (Sui et al., 2013, 2015a, 2015b; but see Schäfer & Frings, 2019). In behavioral research investigating the cognitive origins of self-bias, a quite different picture has emerged, prompting researchers to suggest that stimulus prioritization may originate instead in different underlying mechanisms: including capacity limitations in working memory and response-related processes (e.g., Constable et al., 2019a, 2019b; Falbén et al., 2020; Golubickis et al., 2018; Janczyk et al., 2019; Reuther & Chakravarthi, 2017). What these diverse viewpoints suggest is that, far from representing a unitary phenomenon, stimulus prioritization can be underpinned by a variety of operations. That is, depending upon the task, stimuli, and processing objectives in place, there are multiple routes to the prioritization of material associated with the self.

Consider, for example, classic matching tasks in which geometric shapes are associated with labels pertaining to various persons (Sui et al., 2012). When no other potentially task-relevant information is provided, the most salient feature of the immediate context is the experimentally induced shape-label associations (e.g., self = triangle, friend = square). Unsurprisingly, therefore, participants adopt a task-related strategy that focuses on the self-relevance of the stimuli (I am a triangle), a tactic that is undoubtedly bolstered by the enhanced strength of self-shape (vs. friend-shape) associations in working memory (Reuther & Chakravarthi, 2017). Interestingly, as revealed in the current inquiry, a default preference for self-relevant material also arises when participants are informed that self- and friend-related items are equally likely to be encountered during the task (i.e., Expt. 1). Critically, however, when expectancies about the occurrence of self-related and friend-related items differ, self-relevance is replaced by stimulus prevalence as the most pertinent aspect of the task context. Correspondingly, frequently (vs. infrequently) encountered stimuli are prioritized during shape-label matching. Nevertheless, processing gains are most pronounced for material associated with the self, reflecting the powerful influence that self-relevance exerts during decisional processing (Humphreys & Sui, 2016; Sui & Humphreys, 2015, 2017).

The plasticity of stimulus prioritization also extends to its underlying origins. Rather than stimulus prioritization comprising an exclusively perceptual phenomenon (Humphreys & Sui, 2016; Sui & Humphreys, 2015, 2017), stimulus enhancement is underpinned by operations that are highly sensitive to the prevailing task context. Considering only research that has adopted a DDM analysis to elucidate sources of decisional bias (i.e., stimulus and/or response biases), a complex picture emerges. While, as in the current investigation, prioritization can be driven by differences in the rate of information uptake (i.e., a stimulus bias; Golubickis et al., 2017, 2020), in other settings, bias originates in the evidential requirements of response selection (a response bias; Falbén et al., 2020; Golubickis et al., 2018, 2019). Much like the emergence of stimulus prioritization, of importance are the characteristics of the task context in which bias is assessed. When tasks favor the adoption of egocentric decisional strategies or specific responses are more probable or rewarding than others, stimulus prioritization is underpinned by differences in the evidential requirements of response selection (Falbén et al., 2020; Golubickis et al., 2018, 2019). In contrast, when differences in the strength of associations in working memory influence the ease with which the veracity of shape-label pairings can be established (Ratcliff et al., 2016; Reuther & Chakravarthi, 2017), decisional bias resides in the rate at which information is extracted from stimuli (Golubickis et al., 2017, 2020; Hu et al., 2020; Janczyk et al., 2019). Thus, rather than reflecting the operation of a single underlying process, there are several pathways to stimulus prioritization. As a function of task context and prevailing goal states, optimal decision-making reflects shifts in the priority given to stimulus and response-related operations (Bogacz et al., 2006; Leite & Ratcliff, 2011; White & Poldrack, 2014).

## Conclusion

In a shape-label matching task, here we showed that stimulus prioritization was sensitive to the frequency of stimulus presentation in combination with the personal relevance of the to-be-judged items (cf. Sui et al., 2014). When self- and friend-related items were equally likely to be encountered, a self-prioritization effect was observed. Additionally, in both confirmatory and dis-confirmatory task contexts, stimuli that were encountered frequently (vs. infrequently) were prioritized, an effect that was most pronounced for self-relevant (vs. friend-relevant) items. Underpinning these prioritization effects was variation in the rate of information uptake, such that decisional evidence was extracted more rapidly from self-related than friend-related stimuli. These findings further inform understanding of the emergence and origin of self-prioritization effects during decisional processing.

## Supplementary Information

Below is the link to the electronic supplementary material.

Supplementary file1 (DOCX 16 KB)

## Data Availability

The data generated in the current experiments are available at the Open Science Framework at the following link: osf.io/cj7fp.

## Footnotes

1 It should be noted, however, that a range of non-self-relevant factors, including grammatical distinctiveness, item concreteness, and reward, also elicit stimulus-prioritization effects (e.g., Schäfer et al., 2017; Sui et al., 2012; Wade & Vickery, 2017).

2 Whereas self-prioritization reliably emerges on shape-label matching trials, prior research has yielded mixed evidence for self-related processing gains during nonmatching trials (Frings & Wentura, 2014; Moradi et al., 2015; Reuther & Chakravarthi, 2017; Schäfer et al., 2015, 2016; Sui et al., 2012).

3 Note that in contrast to conventional *p* values, the Bayesian *p* computes the level at which posterior distributions are consistent with the hypothesis of interest (e.g., self > friend). For instance, a Bayesian *p* of .10 is indicative of 90% support for the hypothesis.

## Code availability

Codes are available from the first author on request.
