# Supplementary material for: More or less of me and you: self-relevance augments the effects of item probability on stimulus prioritization
Source: Psychol Res. 2021 Jul 29;86(4):1145–64. doi: 10.1007/s00426-021-01562-x (PMC9090897; doi:10.1007/s00426-021-01562-x)
Supplement: Supplementary file 1 — Supplementary file1 (DOCX 16 KB) [file 426_2021_1562_MOESM1_ESM.docx]

**Supplementary Material**

*Table S1*. Mean reaction time and accuracy as a function of Shape Association and Matching Condition (Expt. 1, Equivalent Context).

Matching Condition Matching Non-Matching

Shape-Association

RT (ms)

self 592 (59) 682 (74)

friend 657 (77) 661 (68)

Accuracy (%)

self 83 (8) 74 (14)

friend 70 (14) 73 (15)

Note. Standard deviations appear within parentheses.

*Table S2*. Mean reaction time and accuracy as a function of Stimulus Frequency, Shape Association, and Matching Condition (Expt. 2, Confirmatory Context).

Matching Condition

Matching Non-Matching

Self- Friend- Self- Friend-

Stimulus Frequency Frequent Frequent Frequent Frequent

Shape-Association

RT (ms)

self 575 (100) 629 (94) 639 (90) 685 (114)

friend 670 (131) 592 (90) 718 (126) 619 (91)

Accuracy (%)

self 90 (10) 74 (18) 84 (13) 70 (16)

friend 60 (20) 79 (14) 64 (17) 81 (13)

Note. Standard deviations appear within parentheses.

*Table S3*. Mean reaction time and accuracy as a function of Stimulus Frequency, Shape Association, and Matching Condition (Expt. 3, Disconfirmatory Context).

Matching Condition

Matching Non-Matching

Self- Friend- Self- Friend-

Stimulus Frequency Frequent Frequent Frequent Frequent

Shape-Association

RT (ms)

self 539 (83) 595 (82) 592 (90) 660 (102)

friend 632 (121) 598 (81) 647 (113) 611 (92)

Accuracy (%)

self 85 (9) 76 (19) 80 (16) 72 (14)

friend 57 (22) 73 (19) 62 (17) 79 (11)

Note. Standard deviations appear within parentheses.
